# Cardiac hemangioma presenting as a primary cardiac tumor

**DOI:** 10.1186/s40959-023-00154-5

**Published:** 2023-01-17

**Authors:** Leart Berdica, Erisa Kola, Daniela Nakuci, Edlira Horjeti, Mehdi Alimehmeti

**Affiliations:** 1 Department of Pathology, Mother Teresa University Hospital Center, Tirana, Albania; 2 Department of Pathology, Gjirokaster Hsopital, Gjirokaster, Albania; 3 Department of Pathology, Vlora Hospital, Vlorë, Albania; 4 Family Medicine, Tirana, Albania; 5 University of Medicine of Tirana, Tirana, Albania

**Keywords:** Cardiac neoplasms, Hemangioma, Myxoma, Left atrium tumor

## Abstract

Cardiac hemangiomas are very rare benign cardiac tumors. They can present at any age and clinical presentation varies according to location and size. We encountered an 87-year-old woman with a left atrial hemangioma clinically diagnosed as cardiac myxoma. Histopathological examination revealed that it was a cavernous-type hemangioma Left atrial hemangiomas, especially those attached to the left atrium wall, may be mistakenly diagnosed as myxomas. Furthermore, a comprehensive review of atrial hemangioma was conducted for the diagnosis and treatment of this uncommon entity.

## Introduction

Cardiac hemangiomas (CH) are rare primary tumors of the heart and constitute only 2.8% of primary cardiac tumors [1]. Microscopically hemangiomas are characterized by benign proliferative endothelial cells lining blood vessels with increased vascularization [2]. The vascular channels are lined by endothelial cells with moderately pleomorphic, sometimes atypical nuclei and focal tuft formation. Mitoses are extremely rare. Histopathologic features of CH are identical to those of hemangiomas elsewhere in the body. Based on the predominant type of the proliferating vessels, hemangiomas are classified into cavernous, capillary and arteriovenous types. Cardiac hemangiomas are benign tumors and lack the ability to metastasize, so they usually grow at a slower rate than malignant tumors. Moreover, they are well circumscribed by an outer surface. However, case reports of hemangiomas invading the conductive tissue of the heart suggest that they are unlike the usual benign tumors. Therefore, despite its histopathologic benignity, CH is regarded as clinically dangerous, owing to the risk of life-threatening complications like syncope, stroke, and even sudden death. Owing to its rarity, to our knowledge this disorder is derived from case reports, and no comprehensive review or clinical guidelines are available. Therefore, the purpose of this presentation is to provide a comprehensive analysis of CH based on our case and literature review.

## Case presentation

An 87-year-old female was referred to the hospital with complaints of dyspnea, dizziness and weakness for the past twelve months. Symptoms had deteriorated three weeks prior to her visit in the hospital. Physical examination revealed arterial blood pressure 100/80 mmHg, heart rate 120 bpm/min, mild jugular regurgitation, and regular cardiac rhythm without murmurs.

**Fig. 1:**
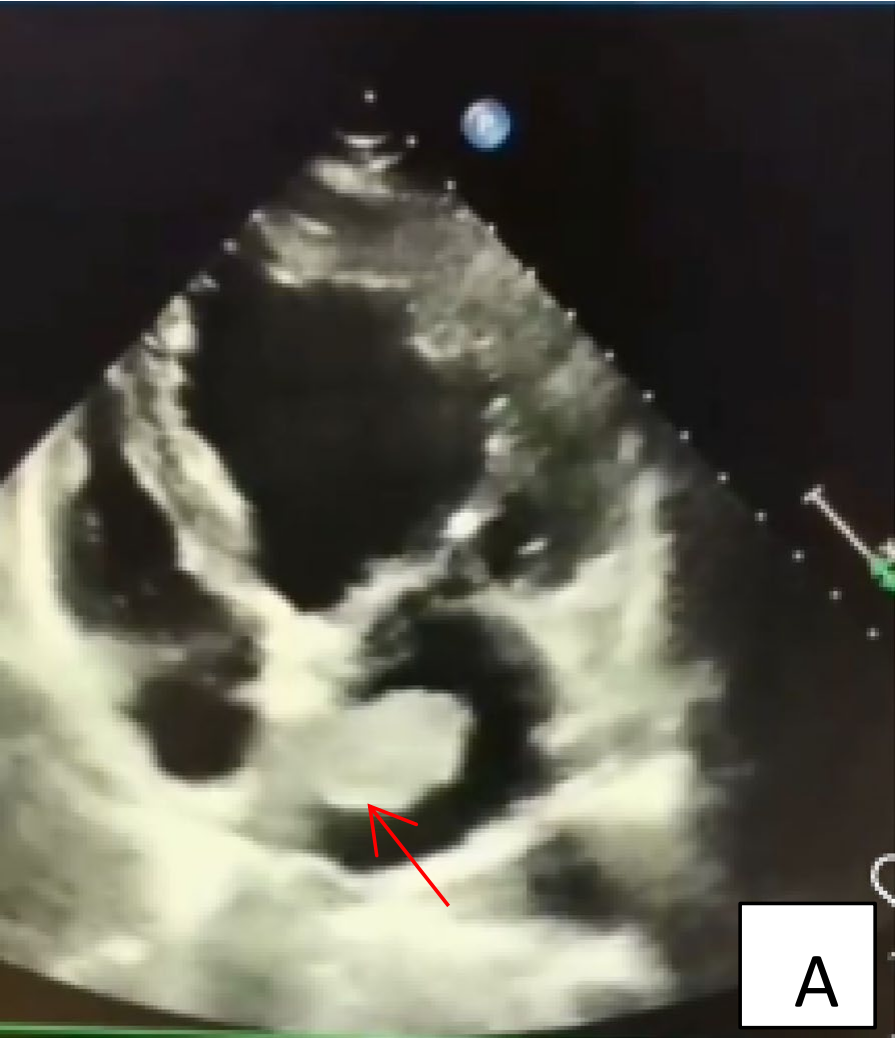
Preoperative echocardiographic findings. Transthoracic four-chamber view echocardiograph of the hemangioma (arrow), showing an irregularly shaped mass attached with a 5 mm- wide stalk to the antero-septal wall of the left atrium

**Fig. 2:**
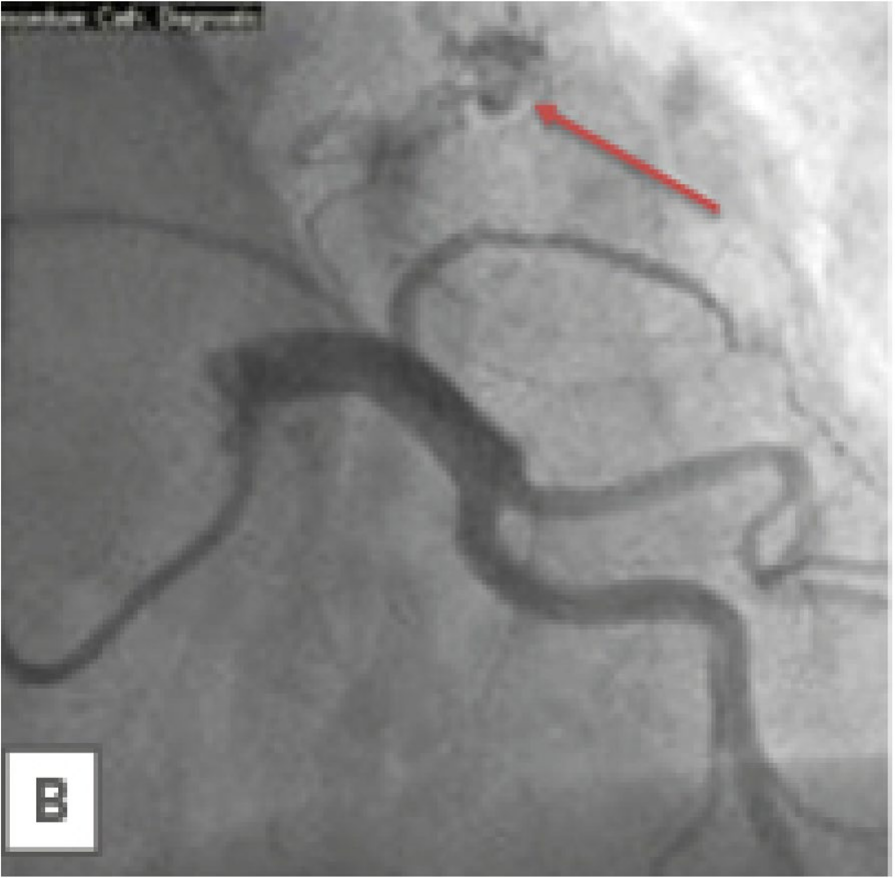
Coronary angiography -demonstrated normal coronary arteries and a vascular blush through the tumor

Her past history was unremarkable. A transthoracic echocardiogram was performed, which revealed an isoechogenic mass (2.1 × 1.5 cm) located in the left atrium. There was no pericardial effusion (Figs. 1 and 2).

After completing the examinations since there was no significant compression of other cardiac and vascular structures, a provisional diagnosis of left atrial cardiac myxoma was made, with possible thrombus. She subsequently underwent coronary angiography which demonstrated normal coronary arteries with no presence of stenosis. The next step in management was surgery and excision of the mass for further microscopic evaluation. Under general anesthesia with supine position, median sternotomy was performed. Intraoperatively, we confirmed that the mass was attached to the atrial septum which was consistent with preoperative echocardiography and had a wide sessile implant basis involving the inter-atrial septum wall of the left atrium. Therefore we explored the left atrium under normothermic cardiopulmonary bypass and blood cardioplegia.

**Fig. 3:**
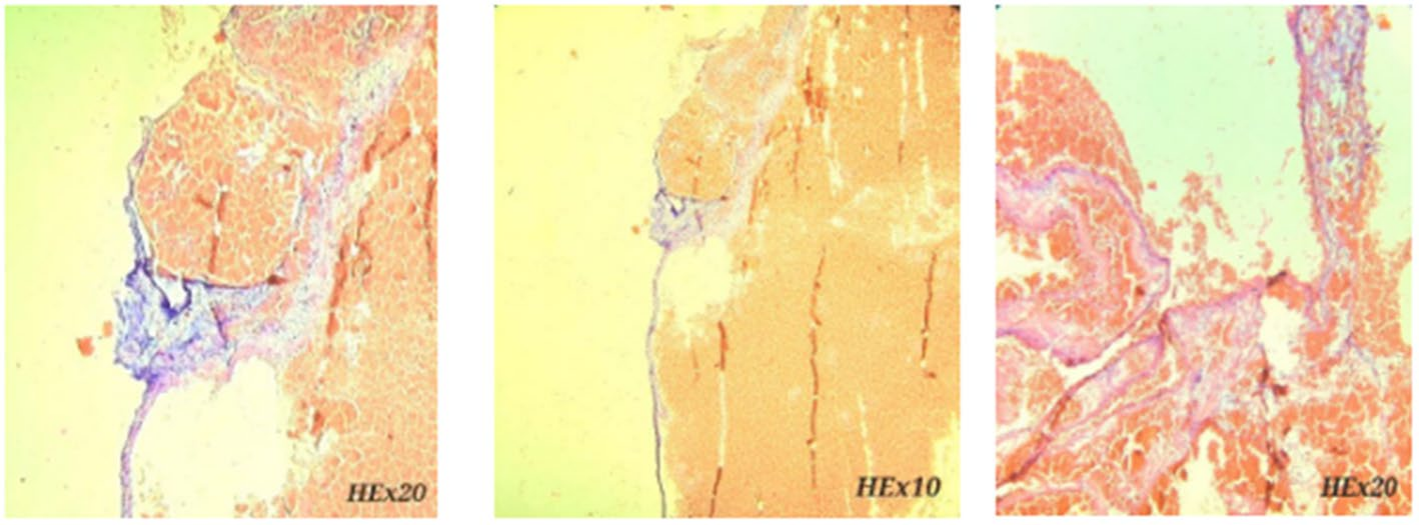
Pathologic findings of left atrial cardiac hemangioma. Hematoxylin and Eosin (H&E) stained sections showed dilated, congested thin walled vascular channels filled with red blood cells and lined with benign endothelial cells, without atypia

The surgical intervention was successful and the cardiac mass was totally removed without affecting the mitral valve and septum. After stopping aortic clamping and cardiac reperfusion, the patient returned to sinus rhythm. Histopathological examination of the excised specimen revealed an encapsulated soft, jelly-like mass measuring 2.2 × 1.5 cm, with a thickened epicardial surface, due to the inflammatory changes. Microscopically, the lesion showed dilation of the engorged vessels comprising multiple thick and thin-walled blood-filled vascular channels in a hyalinized stroma. There was no evidence of atypia or necrosis (Fig. 3).

**Fig. 4:**
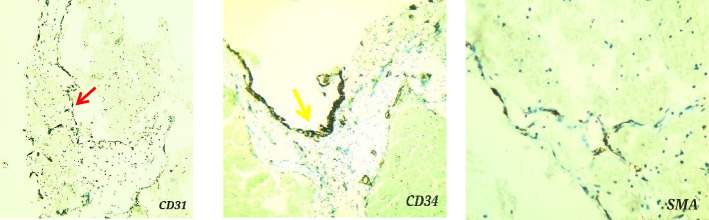
CD31 staining of endothelial cells flooring dilated vessels (red arrow)

Immunohistochemical staining indicated strong immunoreactivity against CD31 and CD34 on endothelial cells lining of tangled capillaries (Fig. 4, red arrow, yellow arrow). The endothelial cells lining the cavernous spaces reacted strongly with CD34 and SMA (Smooth muscle actin).

The pathologic diagnosis based on H&E and immunostains was consistent with cavernous hemangioma of the left atrium. After operation, the patient had an uneventful recovery without any complications.

## Discussion

Hemangiomas are nonmalignant vascular tumors arising predominately in the skin, and less commonly involving intra-cardiac location. These tumors with ambiguous etiology, can be precisely classified via a combination of clinical presentation, histopathologic inspection, radiologic examination including echocardiography, diagnostic imaging (CT, MRI), meticulous surgical resection or autopsy [3, 4]. Histological features of cardiac hemangiomas are similar of extracardiac hemangiomas, composed of vascular engorgement channels bordered by benign endothelial cells. Cardiac hemangioma occurs anywhere in the heart and could be located in the pericardium, endocardium, and myocardium. According to pathology, a cardiac hemangioma can be classified into simple cavernous hemangioma, simple capillary hemangioma, arteriovenous hemangioma, and mixed type. In our literature review of cardiac hemangioma, cavernous hemangioma is the most common pathological type, which accounted for 58.5%, followed by simple capillary hemangioma, accounting for 9.2% [5] (Table 1).

**Table 1:** Cardiac tumors classification

Hemangioma: 1. Capillary; 2. Cavernous; 3. Arteriovenous Hemangiomas
Myxoma
Fibromyoma
Lipoma
Leiomyoma
Mesothelioma
Papillary Fibroelastoma

Asymptomatic CHs are also discovered during evaluation of other heart conditions and predominately showing insight to other diagnosis [6]. Papillary fibroelastoma, a tumor arisen from endocardium cells extended adjacent to the atrioventricular valves, is believed to have a predilection for elderly compared to children, due to mechanical stress of calcified valves. Fibromas are usually solitary masses, which originate from connecting tissues and usually are located into interventricular septum [7]. The biopsy shows unencapsulated fibrous tissue proliferation, with sparse capillaries in the center easily invading structure nearby. Also, other rare tumors are reported including: arteriovenous malformation tumors of the heart, cirsoid aneurism with dysplastic malformation of arteries and veins, lipoma, myxoma, leiomyoma and mesothelioma which are often an incidental finding [8]. According to our literature review, most of the cases reported were diagnosed during symptomatic clinical investigations; nevertheless some of them were accidental findings in non-complaint patients. The range of symptoms associated with CH, depend on the tumor’s location and evolution, including compression and infiltration of the adjacent compartment, rupture, bleeding, embolization and infection [8–10]. Atrial hemangiomas, especially those attached to the septum, may be mistakenly clinically and radiological diagnosed as myxomas. However, there are no myxoma cells or ring structures in cardiac hemangiomas, and cellular areas with numerous capillaries are usually present [6]. Some low-grade angiosarcomas may be difficult to be distinguished from hemangiomas. However, lack of mitotic activity, cellular pleomorphism, necrosis, and cellularity can discern a hemangioma from an angiosarcoma [8]. Hemangiomas can also be differentiated from left ventricular thrombi based on their shape, boundary, echogenicity, vascularity, and including cardiac medical history [11, 12]. CHs are commonly detected as hyperechoic malformations in ultrasound examination. Routine two-dimensional echocardiography can determine the size, location, activity, and compression of the tumor [13]. The features of cardiac tumor manifest as clear boundaries, regular figures, and uniform density. In addition, Color Doppler echocardiography detects blood flow signals in the tumor which are often accompanied by an enlargement of the heart chamber, valve insufficiency, and different degrees of valve regurgitation [4]. However, due to the lack of threedimensionality, two-dimensional echocardiography often mistakes the bulge caused by compression of the extraluminal mass on the heart muscle as an intracardiac mass. Preoperative misdiagnosis for cardiac hemangiomas was common according to our literature review [9]. Histopathologic examination is essential for final diagnosis. Follow-up is recommended to identify any recurrence, even though the rate of recurrence is unknown. 

## Conclusions

Cardiac hemangioma is a rare benign slow growing tumor that occurs in the middle-aged and elderly population. Echocardiography is an accurate, noninvasive method that could contribute to the diagnosis of this disease; however, the final diagnosis still depends on histopathologic examination. Surgical resection of the tumor is the treatment of choice and the prognosis is usually favorable. In our case the patient had a satisfactory outcome after the resection and is under follow-up for the possibility of recurrence.
